# Utilization of Peptidoglycans from Lactic Acid Bacterial Cell Walls for the Mitigation of Acrylamide and 5-Hydroxymethylfurfural

**DOI:** 10.3390/toxics12060380

**Published:** 2024-05-23

**Authors:** Hui Yang, Xue Zhang, Yadong Zhu, Bo Zhang, Junfeng Fan, Hongfei Zhao, Bolin Zhang

**Affiliations:** Beijing Key Laboratory of Forest Food Processing and Safety, College of Biological Science & Biotechnology, Beijing Forestry University, Beijing 100083, China; yanghui_h@bjfu.edu.cn (H.Y.); zhangxue0166@bjfu.edu.cn (X.Z.); zhuyadong888@bjfu.edu.cn (Y.Z.); zhangbo@btbu.edu.cn (B.Z.); fanjunfeng@bjfu.edu.cn (J.F.)

**Keywords:** acrylamide, 5-hydroxymethylfurfural, lactic acid bacteria, peptidoglycan, binding

## Abstract

Acrylamide (AA) and 5-hydroxymethylfurfural (HMF), which are potentially carcinogenic to humans, are often produced during the hot processing of foods. This study first used a molecular docking model to simulate the binding behavior of four lactic acid bacteria peptidoglycans (PGNs) to AA/HMF, and the binding rate of LAB-based PGNs to AA/HMF was evaluated in vitro. In silico results show that interaction energy is the driving force responsible for the adsorption of LAB-derived PGNs to AA/HMF. In vitro results showed that the PGN of *B. lactis* B1-04 bound the most AA (28.7%) and HMF (48.0%), followed by *L. acidophilus* NCFM, *B. breve* CICC 6079, and *L. plantarum* CICC 22135. Moreover, an AA/HMF-bound layer on the cell surface of *B. lactis* B1-04 was observed via AFM and SEM due to adsorption. XPS analysis indicated the removal rate of AA/HMF by selected strains was positively correlated with the proportion of C-O, C=O, and N-H groups of PGNs. The atoms O1, O2, O3, O4, N1, N2, N3, H1, and H2 are involved in the adsorption of LAB-based PGNs to AA/HMF. Thus, the PGNs derived from these four *Lactobacillus* strains can be regarded as natural adsorbents for the binding of AA/HMF.

## 1. Introduction

Heat treatment is one of the most critical processing methods for improving food quality and prolonging the shelf life of food products [1]. However, food heating causes several chemical reactions, including the Maillard reaction (MR). It is well known that the MR, as one of the most common non-enzymatic browning reactions during food heat treatment, can have effects on food quality, mainly related to the aroma, flavor, and color of food [2]. The MR produces unwanted compounds, especially acrylamide (AA) and 5-hydroxymethylfurfural (HMF) [3]. AA is a class of compounds that has attracted much attention in recent years because of its neurotoxicity, genotoxicity, reproductive toxicity, immunotoxicity, and carcinogenicity [4,5]. It is classified as a class 2A carcinogen by the International Agency for Research on Cancer (IARC). Additionally, HMF can be directly transformed into 5-sulfoylmethylfurfural with potent carcinogenicity and genotoxicity through chlorination and vulcanization in vivo; thus, HMF is known as an endogenous food pollutant with potential safety risks [6]. AA/HMF is widely found in everyday foods such as bread, biscuits, breakfast cereals, and crisps. In 2010, the average AA concentration in biscuits detected by the EU reached 1.337 mg/kg, far exceeding the limit value of 0.350 mg/kg set by the new EU Act for biscuits and cookies [7,8]. According to preliminary estimates, the upper limit of HMF intake per person in the population as assessed by the European Food Safety Authority is 1.6 mg/d [9,10], which is much higher than the upper limit of 640 μg per person per day obtained by the Joint Expert Committee on Food additives through a large number of acute and subacute animal toxicology experiments [11,12].

In recent years, investigations into the formation mechanism and detection methods of AA/HMF in hot processed food have been conducted, and the influence of different raw materials and processing technologies on the two chemicals has been explored [13,14,15]. Interestingly, a reduction in the level of AA in biscuits might be attributed to fermentation by selected LAB strains [15]. However, acid production by LAB fermentation lowers the pH of the reaction system and promotes HMF formation. Because of the difference in pH between the AA/HMF reaction systems, the difficulty of synchronous inhibition increases.

Cultures of lactic acid bacteria, particularly *Lactobacillus* and *Bifidobacterium*, can bind to dietary carcinogens [16]. It has been proposed that PGN of the bacterial cell wall is regarded as an essential component binding carcinogens [17]. Lahtinen et al. found that exogenous polysaccharides and cell wall proteins did not play a role in the process of AFB1 binding, but cell wall PGN or compounds closely bound to PGN did bind AFB1 [18]. A recent study showed that the PGN from *L. acidophilus* NCFM was the main component binding DBP, and the specific groups C-O, N-H, O-H, and C-N were the main binding sites [19]. Goyal et al. revealed the importance and integrity of PGN in the adsorption of DEHP and found that the functional groups C=O, N-H, C-N, and C-O played major roles in adsorption [20]. Similarly, the PGN in the cell wall of LAB strains has been proposed to be the primary mechanism for reducing AA [21]. A similar study found that LAB-based PGN played a major role in binding AA [22], and the specific groups C-O, C=O, and N-H from LAB were the main functional units responsible for AA adsorption by *L. acidophilus* and *B. breve* [23]. Moreover, the carbohydrate and specific amino acids from the cell wall PGN were significantly correlated with the adsorption rate of AA, and the specific groups C-O, C=O, and N-H from LAB-based PGN were the main functional units responsible for AA adsorption by LAB-based PGN [24]. In summary, many studies have shown that some functional groups from the PGN of the LAB cell wall play a major role in adsorption [22,24,25], however, no reports have confirmed the adsorption of AA/HMF by LAB-based PGN.

Therefore, this study firstly predicted the binding site of LAB-based PGN and AA/HMF using in silico calculation of their interaction energy via a molecular docking model. Morphological changes in *Bifidobacterium lactis* B1-04 cells upon AA/HMF binding were observed. Spectral analysis was used to detect the adsorption process of functional groups on PGN towards AA/HMF. Furthermore, the relationship between the adsorption rate of different strains and their PGN-specific groups was analyzed using X-ray photoelectron spectroscopy (XPS). This study aimed to analyze the cell wall peptidoglycan behavior in binding AA/HMF among selected LAB strains.

## 2. Materials and Methods

### 2.1. Chemicals

Standard AA (>99%) was purchased from Aladdin Biochemical Technology Co., Ltd. (Shanghai, China), and standard HMF (>99%) was obtained from Source Leaf Biotechnology Co., Ltd. (Shanghai, China). Methanol and acetonitrile (HPLC grade) were purchased from Thermo Fisher Scientific Co., Ltd. (Waltham, MA, USA). Trichloroacetic acid, trichloromethane, pancreatic enzymes, and neutral protease were obtained from GIBCO Co., Ltd. (Grand Island, NY, USA). 

### 2.2. Bacterial Strains and Culture Cultivation

Freeze-dried powders of *Bifidobacterium breve* CICC 6079 and *Lactobacillus plantarum* CICC 22135 were obtained from the China Center of Industrial Culture Collection (CICC), China. *Lactobacillus acidophilus* NCFM and *Bifidobacterium lactis* B1-04 were donated by DuPont (Shanghai, China). Firstly, all strains were activated in MRS broth in anaerobic tanks (Oxoid, Thermo Fisher Scientific Inc., Waltham, MA, USA), and two subcultures with a 4% inoculum in 300 mL MRS broth incubated at 37 °C for 18 h were performed before use. Then, the LAB cells harvested by centrifugation (6000× *g*, 15 min, 4 °C), after being washed with sterile water, were mixed with 30 mL deionized water for PGN extraction.

### 2.3. Preparation of LAB-Based PGN

The preparation of LAB-based PGN was carried out according to the method of Wu, Pan, Guo, and Zeng (2013), with modification [26,27]. Briefly, the LAB cell suspensions prepared as per Section 2.2 were centrifugated (6000× *g*, 10 min, 4 °C) to collect the precipitations. Then, the sediments, after being mixed with ten-fold volumes of 10% trichloroacetic acid (TCA), were heated and stirred in boiling water at 100 °C for 20 min for lytic cell death. The solution, after being cooled to 4 °C, was centrifuged (6000× *g*, 10 min, 4 °C) to obtain precipitations. The precipitations, after being washed twice and mixed with two-fold volumes of chloroform–methanol (1:2 in *V*:*V*), were held at 4 °C to remove lipids from the cell suspension. After a 6 h treatment, the suspensions were centrifuged again (6000× *g*, 10 min, 4 °C). Next, the prepared sediments were washed with sterile water three times and then dissolved with isovolumetric 0.1 N Tris-HCl (pH 7.6) containing trypsin (3 mg /mL) and neutral protease (10 mg/mL) at 37 °C overnight to remove unwanted proteins from cell surfaces. The solutions were centrifuged (11,000× *g*) for 20 min, and their sediments were treated with sterile water three or four times until the enzyme-like particles disappeared. The remains extracted as LAB-based PGNs were freeze-dried for further study. 

### 2.4. AA/HMF Binding Assay 

A total of 4.0 mg of AA/HMF was dissolved in 1.0 mL sterilized water to prepare a working solution, in which the AA/HMF concentration was 4.0 mg/mL. The 1.0 mL working solution of AA/HMF (4 mg/mL) was mixed with 5.0 mg LAB-based PGN of 4 tested strains and equivalent quality bacterial powder, and then the mixtures were co-cultured at 37 °C for 2 h, 4 h, 6 h, 8 h, and 10 h to detect the adsorption of the PGNs to AA/HMF. The level of AA/HMF was measured using an HPLC system (LC-20AT; Shimadzu Co., Ltd., Kyoto, Japan) equipped with a ZORBAX SB-C18 column (4.6 × 250 mm; 5 μm, Agilent, Santa Clara, CA, USA). All tested samples were filtered through a filter membrane (0.22 mm) before the experiment. The injection volume of each sample was 20 mL, and HPLC grade acetonitrile/water (90:10, *V*:*V*) or methanol/water (90:10, *V*:*V*) was used as the mobile phase with a flow rate of 1 mL/min at 45 °C/25 °C. In this assay, the detection wavelength was 205 nm/283 nm. A positive (sterile H_2_O + AA/HMF) control was included in all experiments. The percentage of binding AA/HMF by the PGNs was calculated according to Equations (1) and (2), respectively.

AA binding rate (%) = (1 − AA peak area of sample/AA peak area of positive control) × 100%(1)

HMF binding rate (%) = (1 − HMF peak area of sample/HMF peak area of positive control) × 100%(2)

### 2.5. Removal of AA/HMF by the PGN of Strain B. lactis B1-04

#### 2.5.1. Morphological Observation of *B. lactis* B1-04 Binding AA/HMF

Two-milligram freeze-dried powders of *B. lactis* B1-04 cells with or without binding AA/HMF were observed by AFM (Atomic Force Microscopy, Bruker Dimension Icon, Bruker, Germany) and SEM (Scanning Electron Microscope, SU8010, HITACHI, Kyoto, Japan). For SEM observation, all dried samples of *B. lactis* B1-04 cells were adhered to the disc sprayed with gold, which was used to increase the conductivity of the sample with an acceleration voltage of 5 kV and a magnification of 7.7 mm × 20.0 k for the micrographs. For AFM observation, the powders of *B. lactis* B1-04 cells, after being dissolved in 1.0 mL water, were shaken very well. Then, the drops were placed in the center of the mica sheet and left to dry until the bacterial cells were fixed. *B. lactis* B1-04 cells with or without binding AA/HMF on the surface of the mica sheet were observed under an atomic force microscope with the probe specification of RFESPA-75 and a scanning speed of 1.0 Hz.

#### 2.5.2. XRD Analysis of the PGN of Strain B1-04 Binding AA/HMF

Two milligrams of freeze-dried PGN of strain B1-04 with or without binding AA/HMF was diffracted by an XRD (X-ray diffractometer, Rigaku Ultima IV, Japan) equipped with Cu ka radiation monochromated by a single graphite crystal, at 40 kV and 40 mA. The diffraction peak was used to describe the crystal structure of the different treatments, indicating the interaction between the PGN of the *B. lactis* B1-04 cell wall and AA/HMF [28].

#### 2.5.3. FTIR Analysis of the PGN of Strain B1-04 Binding AA/HMF 

FTIR (Fourier Transform Infrared Spectrometer, Thermo Scientific Nicolet iS50, Waltham, MA, USA) analysis was carried out to identify the intensity of the absorption spectra directly linked to the molecular composition of the chemical groups of LAB-based PGN. A total of 2.0 mg of freeze-dried PGN of *B. lactis* B1-04 with or without binding AA/HMF was mixed with KBr powder in a ratio of 100:1 and ground in an agate mortar. Then, each sample was pressed into a transparent disc for FTIR detection. The FTIR spectral range was 4000 to 400 cm^−1^ with a measured temperature of 25 °C.

### 2.6. Adsorption Action of the PGNs from Selected LAB Strains to AA/HMF

#### 2.6.1. Construction of the LAB-Based PGN Molecules and AA/HMF Molecular Models

Normally, the PGN scaffold of the LAB cell wall is a repeating unit that consists of N-acetylglucosamine (NAG) and (NAG)-N-acetylmuramic (NAM) disaccharide [NAG (β-1,4)-NAM] having a peptide attached to the D-lactyl moiety of each NAM [29]. Data on the accurate structures of the LAB-based PGNs from our selected four strains have already been described by HPLC, NMR, FTIR, MALDI-TOF/TOF MS, and amino acid analysis. For *B. lactis*, its PGN molecular scaffold consists of NAG-(β-1,4)-NAM-L-Ala-D-Glu-L-Orn-D-Ala, and each NAG-NAM repetition unit is interconnected by an L-Orn-L-Ser-L-Ala-L-Thr-L-Ala peptide chain [30,31]. For *B. breve*, its PGN comprises NAG-(β-1,4)-NAM-L-Ala-D-Glu-L-Lys-D-Ala, and each NAG-NAM repetition unit is linked by an L-Lys-Gly peptide chain [32]. For *L. acidophilus*, its PGN is composed of NAG-(β-1,4)-NAM-L-Ala-D-Glu-L-Lys-D-Ala, and each NAG-NAM repetition unit is tied by an L-Lys-D-Asp peptide chain [26,33]. The PGN of *L. plantarum* consists of NAG-(β-1,4)-NAM-L-Ala-D-Glu-m-Dpm-D-Ala, and each NAG-NAM repetition unit is connected by an m-Dpm direct chain [30,31]. In most cases, the bisdisaccharide peptide dimer represented the maximum fraction of LAB-based PGN, so it is feasible to visualize a PGN scaffold of the LAB cell wall with a bisdisaccharide peptide dimer [33]. 

To explore the differences in the binding ability of the four tested LAB strains to AA/HMF, the three-dimensional structures of LAB-based PGN plus AA/HMF were established using the “Visualizer” module of the molecular simulation model (MS). The interaction forces and fractional free volume (FFV) between the selected LAB-based PGNs and AA/HMF were calculated via the modules “Visualizer”, “Amorphous Cell”, and “Forcite” from Materials Studio19.0 (Accelrys Software Inc., San Diego, CA, USA), and the force field was COMPASS II and DREIDING. Theoretically, the adsorption capacity of each tested strain to AA/HMF could be predicated by the interaction forces and FFV. 

#### 2.6.2. Calculation of the Interaction Energy

A packing model composed of AA/HMF and PGN repeat units was constructed in the “Amorphous Cells” module, with an initial density of 1 g/cm^3^. The packing model was subsequently optimized so that the annealing procedure was heated from 300 K to 500 K at an interval of 30 K and then cooled. For each temperature interval, 5 ps NPT dynamics were designed to maintain the equilibrium status of the simulation system. Afterward, further 20 ps NPT and 20 ps NVT molecular dynamic simulations were performed at 298 K, and the atomic trajectory was recorded for the subsequent analysis. We selected the lowest potential energy and used the DREIDING force field to calculate the interaction energy of the simulated system. The interaction energy between AA/HMF and PGN designated by ΔE_A_/△E_H_(kcal/mol) could be calculated according to thermodynamic Equations (3) and (4).

△E_A_ = E_PGN+AA_ − (E_AA_ + E_PGN_) (3)

△E_H_ = E_PGN+HMF_ − (E_HMF_ + E_PGN_) (4)

where E_PGN+AA_/E_PGN+HMF_ stands for the total energy of the system; E_PGN_ indicates the energy of the isolated PGN molecule; and E_AA_/E_HMF_ represents the energy of AA/HMF molecules. 

#### 2.6.3. Calculation of Fractional Free Volume (FFV)

FFV is the movement of molecular chains and thus can always be used to predict the diffusion behavior of a polymer–solvent mixture. After completing the anneal operation (as seen in Section 2.6.2), the minimum potential energy of the simulation system, expressed as FFV, was calculated to show the stability of LAB-based PGN binding AA/HMF. FFV was calculated using Equation (5).

FFV (%) = Free volume/(Free volume + Occupied volume) × 100%(5)

Free volume stands for the volume of space not occupied by matter; occupied volume indicates the volume occupied by polymers and small molecules.

#### 2.6.4. Calculation of Radial Distribution Function (RDF)

RDF, defined as the probability of space distribution of other particles in given coordinates of one particle, can often be used to analyze the interaction between the “set atoms” [34,35]. Theoretically, the higher and sharper the peaks of the “set atoms”, the more the “set atoms” are closely related. In our case, we selected the atoms having a strong interacting force between LAB-based PGN and AA/HMF as the “set atoms” to search for the potential binding sites. The estimated RDFs of the “set atoms” by the module FORCITE could be used to produce the images containing the peaks of LAB-based PGN and AA or LAB-based PGN and HMF. Potential binding sites between LAB-based PGN and AA/HMF would be identified by comparing the peak shapes of the “set atoms”.

### 2.7. Correlation between the Specific Groups of LAB-Based PGN and AA/HMF Binding 

XPS (X-ray photoelectron spectroscopy), referring to the energy distribution of photoelectrons and Auger electrons emitted from the surface of a sample by X-ray photon irradiation, can be used to determine the chemical properties of the bacterial cell surface [36]. Thus, the relative contents of C, N, and O functional groups from the LAB-based PGN of each tested strain were determined according to the method of Ma et al., with modification [37]. Briefly, 2.0 mg of freeze-dried PGN of the four selected strains was placed into an XPS (Thermo Scientific, USA) chamber with excitation source AI-Kα (energy 1486.6 eV) for determining the proportion of XPS C1s, N1s, and O1s peaks. When the pressure of the sample chamber was less than 2.0 × 10^−7^ mbar, the LAB-based PGN sample was sent to the analysis chamber with a spot size of 400 μm plus a working voltage of 12 kV and a filament current of 6 mA. The full-spectrum scan used a passing energy of 150 eV and a step size of 1 eV. The narrow-spectrum scanning energy was 50 eV, and the step size was 0.1 eV. Next, XPS C1s, N1s, and O1s spectra were fitted to quantitatively analyze the relative content of specific groups from the selected LAB-based PGN using Advantage 5.9 software.

### 2.8. Statistical Analysis

All experimental treatments were carried out in triplicate, and the results were presented as the mean ± standard deviation (SD). Data analysis was performed by the variance (ANOVA) *t*-test using the SPSS 27.0.1 statistical analysis package. Significant differences were considered to be at *p*-values < 0.05. Origin 2021 software is used to generate graphs.

## 3. Results

### 3.1. AA/HMF Binding Assay

**Figure 1:**
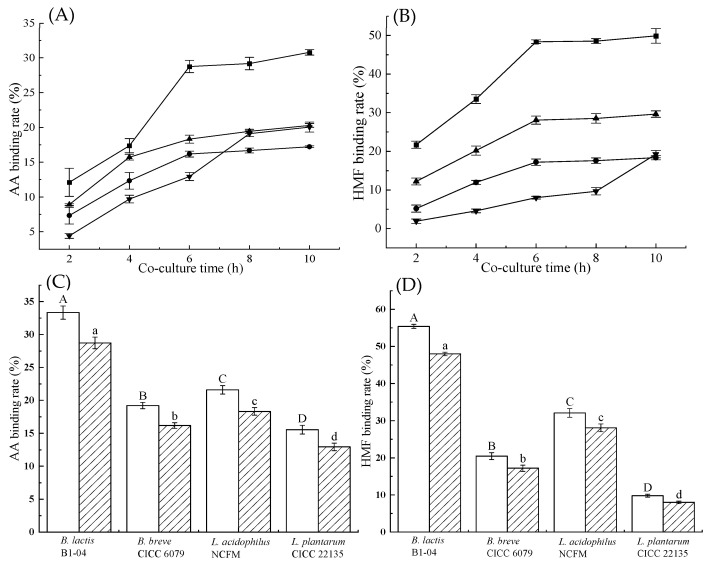
(**A**,**B**) The effects of different co-culture times on the binding rate of the four selected LAB-based PGNs to AA/HMF: 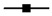 *B. lactis* B1-04; 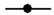 *B. breve* CICC 6079; 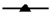 *L. acidophilus* NCFM; 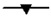 *L. plantarum* CICC 22135. (**C**,**D**) The binding rate of the four selected strains and their PGNs to AA/HMF under the condition of co-culture for 6 h; bars with different letters are statistically different from each other (*p* < 0.05). 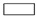 the binding rate of strains; 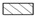 the binding rate of PGNs.

As shown in Figure 1A,B, the LAB-based PGNs of the tested four strains showed varying ability to bind AA/HMF, depending largely on the selected strains. The highest percentage was 28.7% for *B. lactis* B1-04 PGN to bind AA, and 48.0% for *B. lactis* B1-04 PGN to bind HMF, followed by the PGNs of *L. acidophilus* NCFM, *B. breve* CICC 6079, and *L. plantarum* CICC 22135. Moreover, it was seen that the whole cells of the tested four strains showed the same trend in binding AA/HMF as their PGNs did (see Figure 1C,D). The binding capacity of the selected four strains to AA/HMF was found to be highly consistent with the binding rate of their PGNs to the two chemicals. Additionally, incubation time greatly affected the binding capacity of the four selected LAB-based PGNs towards AA/HMF. The percentage of AA/HMF bound by the PGNs of the four tested strains quickly increased during the incubation of 2 h to 6 h and slowly increased after 6 h (*p* < 0.05). These results confirm that LAB-based PGNs, as one of the most important components of the cell wall, play a key role in helping the tested strains to remove AA/HMF.

### 3.2. Morphology and Specific Groups of B. lactis B1-04 Strain Binding AA/HMF

To explore the roles of the LAB-based PGNs in binding AA/HMF, this study firstly selected *B. lactis* B1-04, having the best adsorption capacity, to observe the morphological change of its cells after binding AA/HMF via AFM and SEM, aiming to find which specific groups out of its PGN are involved in the AA/HMF adsorption based on the data of FITR and XRD.

#### 3.2.1. Visualization of the Adsorption of *B. lactis* B1-04 Cells to AA/HMF

**Figure 2:**
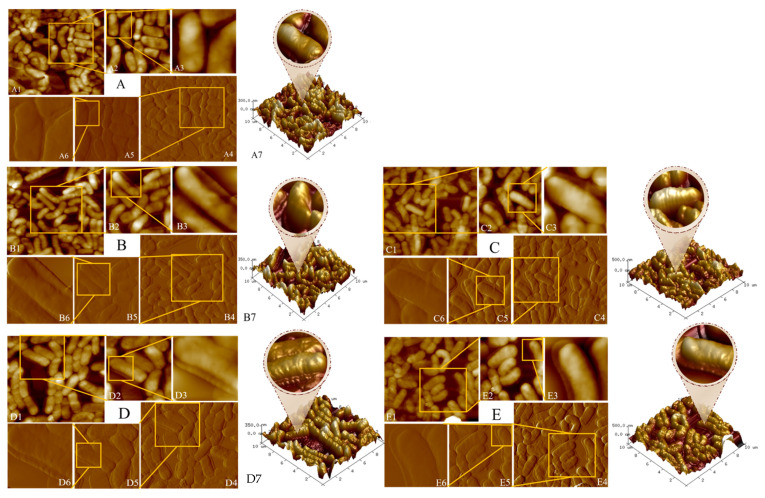
The AFM images of strain *B. lactis* B1-04 under different treatments. (**A**–**E**) *B. lactis* B1-04 architectural features and the *B. lactis* B1-04 cell bound with 4 μg/mL AA, 10 μg/mL AA, 4 μg/mL HMF, and 10 μg/mL HMF. A1, A2, and A3; B1, B2, and B3; C1, C2, and C3; D1, D2, and D3; E1, E2, and E3 shown Height (H) images with scale bars of 10 μm, 5 μm, and 2 μm; A4, A5, and A6; B4, B5, and B6; C4, C5, and C6; D4, D5, and D6; E4, E5, and E6 show Phase (P) images with scale bars of 10 μm, 5 μm, and 2 μm. A7, B7, C7, D7, and E7 represent 3D images.

**Figure 3:**
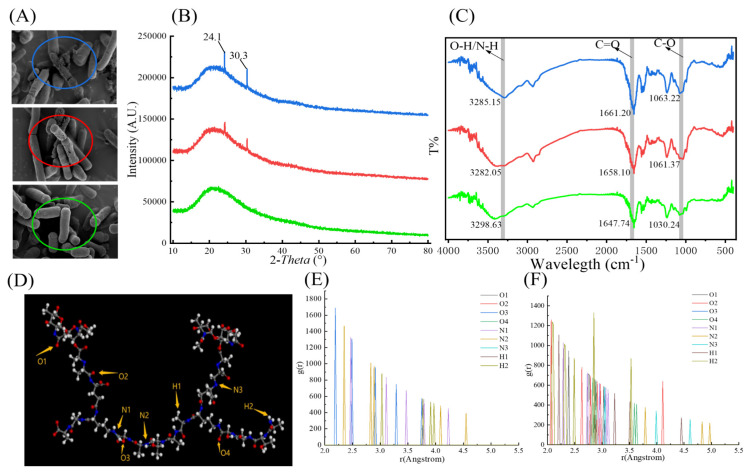
(**A**) SEM of *B. lactis* B1-04 with and without binding AA/HMF (—*B. lactis* B1-04, —*B. lactis* B1-04 bound with AA, —*B. lactis* B1-04 bound with HMF (magnification: 20,000)). (**B**,**C**) XRD and FTIR of *B. lactis* B1-04 PGN bound with AA/HMF (—Control PGN; —PGN + AA; —PGN +HMF). (**D**) *B. lactis* B1-04 PGN model built by “Visualizer modules” of Material studio19.1. Binding sites are observed between *B. lactis* B1-04 PGN and AA/HMF (these positions of binding sites are indicated by the yellow arrows). (**E**,**F**) The radial distribution function of binding sites between *B. lactis* B1-04 PGN and AA/HMF.

As shown in Figure 2 and Figure 3A, the structural characteristics of the *B. lactis* B1-04 cells with or without binding AA/HMF are rod-shaped, with a length of 2.0 to 3.0 μm and a width of 0.6 to 1.0 μm. Before binding AA/HMF, the cell surface of strain B1-04 was seen to not be entirely smooth but rugged, full of folds, concave, and convex. This indicated that the unique morphological surface of bacterial cells might be beneficial for AA/HMF binding. After the binding of *B. lactis* B1-04 cells to AA/HMF, the cell surface, covered with one AA/HMF layer of 50 to 200 nm thickness, became much rougher than the control (see Figure 2B–E). It was clear that the thickened cell surface could be attributed to the greater binding of *B. lactis* B1-04 to AA/HMF.

#### 3.2.2. Specific Groups Responsible for the Binding of *B. lactis* B1-04 PGN to AA/HMF 

FTIR analysis. The average FTIR spectra of *B. lactis* B1-04 PGN with or without binding AA/HMF are shown in Figure 3C. Basically, it was observed that the spectral shape of the PGN from *B. lactis* B1-04 did not change drastically if this strain bound AA/HMF. However, its PGN peaks, with or without binding AA/HMF, have the same shape. The data from Figure 3C show that the characteristic peaks of the *B. lactis* B1-04 PGN spectrum were significantly enhanced after its PGN bound with AA/HMF. This indicated that multiple components of strain B1-04 PGN were involved in the process of AA/HMF binding. Firstly, the absorption peaks of *B. lactis* B1-04 PGN moved from the initial 3298.63 cm^−1^ to 3282.05 cm^−1^ for AA binding and 3285.15 cm^−1^ for HMF binding (see Table 1), indicating that the stretching vibration of O-H and amine N-H of *B. lactis* B1-04 PGN strengthened, and thus the number of hydrogen bonds increased [38]. Secondly, the absorption peaks of strain B1-04 PGN moved from the original 1647.74 cm^−1^ to 1658.10 cm^−1^ for AA binding and 1661.20 cm^−1^ for HMF binding with the increased displacement. This meant that the number of C=O bonds of protein increased due to AA/HMF binding [39]. Thirdly, the absorption peaks of *B. lactis* B1-04 PGN moved from the start-point of 1536.79 cm^−1^ to 1524.31 cm^−1^ for AA binding or 1523.62 cm^−1^ for HMF binding with the decreased displacement, indicating that the number of N-H bonds of amideⅡdecreased due to AA/HMF binding [40]. Finally, the absorption peaks of *B. lactis* B1-04 PGN moved from the initial 1030.24 cm^−1^ to 1061.37 cm^−1^ for AA binding and 1063.22 cm^−1^ for HMF binding with the increased displacement. The results showed that the number of single bonds of carbohydrates such as C-O, C-C, C-O-H, and C-O-C increased due to AA/HMF binding [41]. In summary, FTIR data showed that O-H and N-H bonds of amines, C=O bonds of proteins, N-H bonds of amideⅡ, and single bonds of saccharides (C-O, C-C, C-O-H, C-O-C) are likely the specific groups responsible for the adsorption of strain B1-04 PGN to AA/HMF. 

XRD analysis. The crystal structures of *B. lactis* B1-04 PGN, with or without AA/HMF binding, were affirmed by XRD. As shown in Figure 3B, the configuration of new acute crystallization peaks at 2 Theta of 24.1° and 30.3° appeared after *B. lactis* B1-04 PGN bound AA/HMF. The presence of the new acute crystallization peaks is obviously attributed to the appearance of a powerful interaction within and between molecules that have firm crystallinity [28]. Unlike the PGN not binding AA/HMF, the crystal structure of *B. lactis* B1-04 PGN adsorbing AA/HMF was apparently reinforced, because the interaction between AA/HMF and *B. lactis* B1-04 PGN caused the initial PGN crystal structure to be destroyed, thus forming a stronger crystallinity. Thus, data from XRD further addressed the powerful interaction between AA/HMF and *B. lactis* B1-04 PGN.

RDF analysis. At the same time, the current study used the RDF tool to analyze the binding performance of specific groups from *B. lactis* B1-04 PGN towards AA/HMF. As shown in Figure 3D, the binding sites between *B. lactis* B1-04 PGN and AA/HMF were represented by the atoms O1, O2, O3, O4, N1, N2, N3, H1, and H2. Furthermore, RDF analysis of the main binding sites of *B. lactis* B1-04 PGN towards AA showed that the peaks of the atoms O3, N2, N1, H2, N3, and O2 appeared earlier, leading the peak shapes to be high and sharp. The high and sharp peaks represent possible sites of strain B1-04 PGN to bind AA in the adsorption process. Comparing g (*r*) values showed that the important order of the nine binding sites was O3 > N2 > N1 > H2 > N3 > O2 > O4 > O1 > H1 (see Figure 3E). Meanwhile, RDF analysis of the main binding sites of *B. lactis* B1-04 PGN towards HMF showed that the peaks of the atoms H2, O3, O2, H1, O1, and N1 appeared earlier, leading the peak shapes to be high and sharp. Thus, the atoms H2, O3, O2, H1, O1, and N1 are likely the main binding sites of strain B1-04 PGN towards HMF in the adsorption process. Comparing g (*r*) values showed that the influence order of the nine binding sites was H2 > O3 > O2 > H1 > O1 > N1 > O4 > N3 > N2, as seen in Figure 3F. Clearly, RDF analysis confirmed the key role of the specific groups represented by oxygen, nitrogen, and hydrogen atoms in performing the adsorption of strain B1-04 PGN to AA/HMF.

### 3.3. Adsorption Action of the PGNs from the Selected LAB Strains to AA/HMF

#### 3.3.1. Interaction Energy Analysis of Adsorbed AA/HMF 

**Figure 4:**
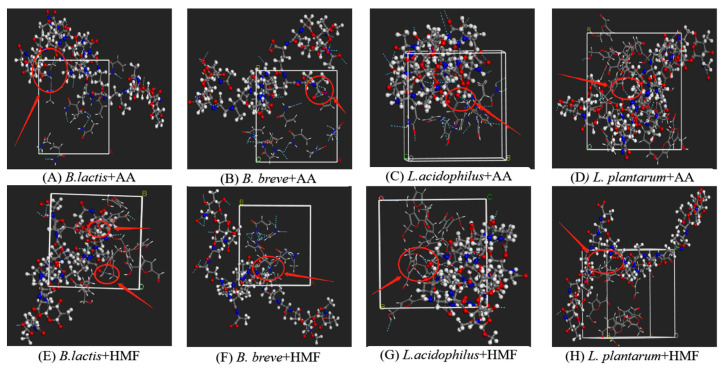
(**A**–**D**) The adsorption model regarding four selected LAB-based PGNs binding with AA. (**E**–**H**) The adsorption model regarding four selected LAB-based PGNs binding with HMF.

Figure 4A to Figure 4H display the molecular visualization of the LAB-based PGNs of the four tested strains binding AA/HMF. Table 1 lists the interaction energy between LAB-based PGNs of the four tested strains and AA/HMF. Regarding the adsorption of the LAB-based PGNs to AA, the interaction energy ranked as △E_A1_ = (247.087 kcal/mol) > △E_A3_ = (222.577 kcal/mol) > △E_A2_ = (200.706 kcal/mol) > △E_A4_ = (182.359 kcal/mol). For the adsorption of LAB-based PGNs to HMF, the interaction energy was △E_H1_ = (293.293 kcal/mol) > △E_H3_ = (273.21 kcal/mol) > △E_H2_= (258.669 kcal/mol) > △E_H4_ = (247.675 kcal/mol). Theoretically, the higher the interaction energy the more the LAB-based PGN bound AA or HMF. Clearly, it could be speculated that *B. lactis* B1-04 would bind the most AA or HMF, followed by *L. acidophilus* NCFM, *B. breve* CICC 6079, and *L. plantarum* CICC 22135, in terms of their interaction energy. In summary, the adsorption of LAB-based PGNs to AA/HMF is driven by their interaction energy. 

#### 3.3.2. FFV Analysis of Adsorbed AA/HMF 

**Figure 5:**
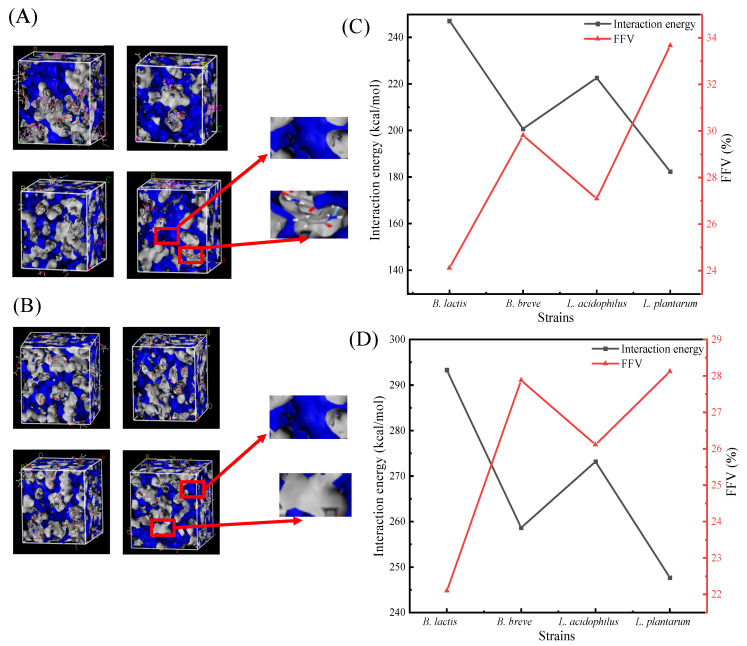
(**A**,**B**) Three-dimensional representation of FFV regarding four selected LAB-based PGNs binding with AA/HMF, where the blue area represents the FFV and the gray area is the occupied capacity. (**C**,**D**) The interaction energy and FFV of four selected LAB-based PGNs binding with AA/HMF.

A 3D representation of FFV (fractional free volume) showing four selected LAB-based PGNs binding with AA/HMF is presented in Figure 5A,B, in which the blue area represents the free volume and the gray area represents the occupied capacity. Regarding FFV values of the four selected LAB-based PGNs binding with AA/HMF, the size of FFV is strain-specific. The lowest FFV values were observed in the binding of *B. lactis* B1-04 PGN towards AA/HMF. The FFV was 24.12% for *B. lactis* B1-04 PGN towards AA, and 22.10% for its PGN towards HMF. FFV values ranked as *L. plantarum* CICC 22135 > *B. breve* CICC 6079 > *L. acidophilus* NCFM > *B. lactis* B1-04, as shown in Figure 5C,D (detailed information can be found in Table 2). It is interesting to note that the lower the FFV values, the larger the interaction energy between the selected LAB-based PGNs and AA/HMF, producing stronger binding efficiency. Similar results were confirmed by Zhao et al. (2021), who presumed that the interaction energy was greater, resulting in the proximity of molecules to each other and a smaller FFV [19]. Clearly, the high FFV value means the low adsorption of LAB-based PGN towards AA/HMF.

### 3.4. Effect of Relative Content of Specific Groups on LAB-Based PGN on AA/HMF Binding

#### 3.4.1. The Full Spectrum Analysis of XPS

**Figure 6:**
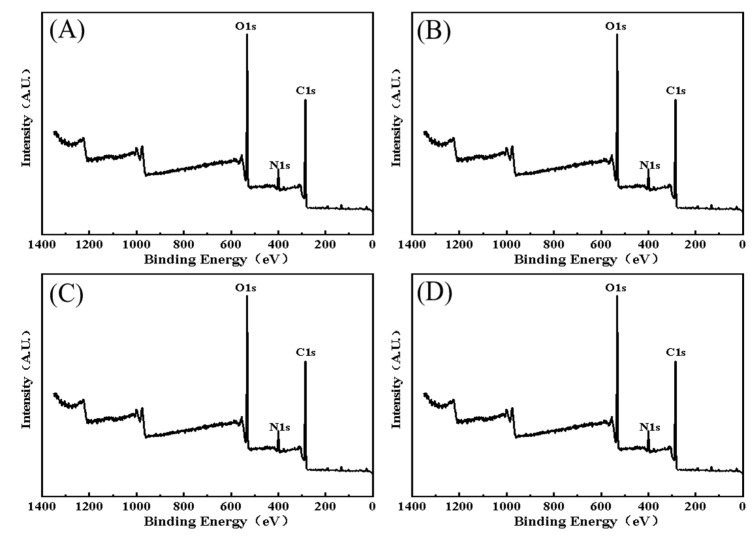
(**A**–**D**) XPS spectra of four selected LAB-based PGNs. ((**A**): *B. lactis* B1-04; (**B**): *B. breve* CICC 6079; (**C**): *L. acidophilus* NCFM; (**D**): *L. plantarum* CICC 22135).

The full spectra of the LAB-based PGNs from the four tested strains were detected by XPS, as indicated in Figure 6. The prominent spectral peaks for the LAB-based PGNs are carbon, nitrogen, and oxygen. The single spectrum peak of XPS from the LAB-based PGNs, as seen in Figure 5, found that the specific group contents of the LAB-based PGNs from the four selected bacteria varied from strain to strain, described as follows.

#### 3.4.2. C Spectrum

**Figure 7:**
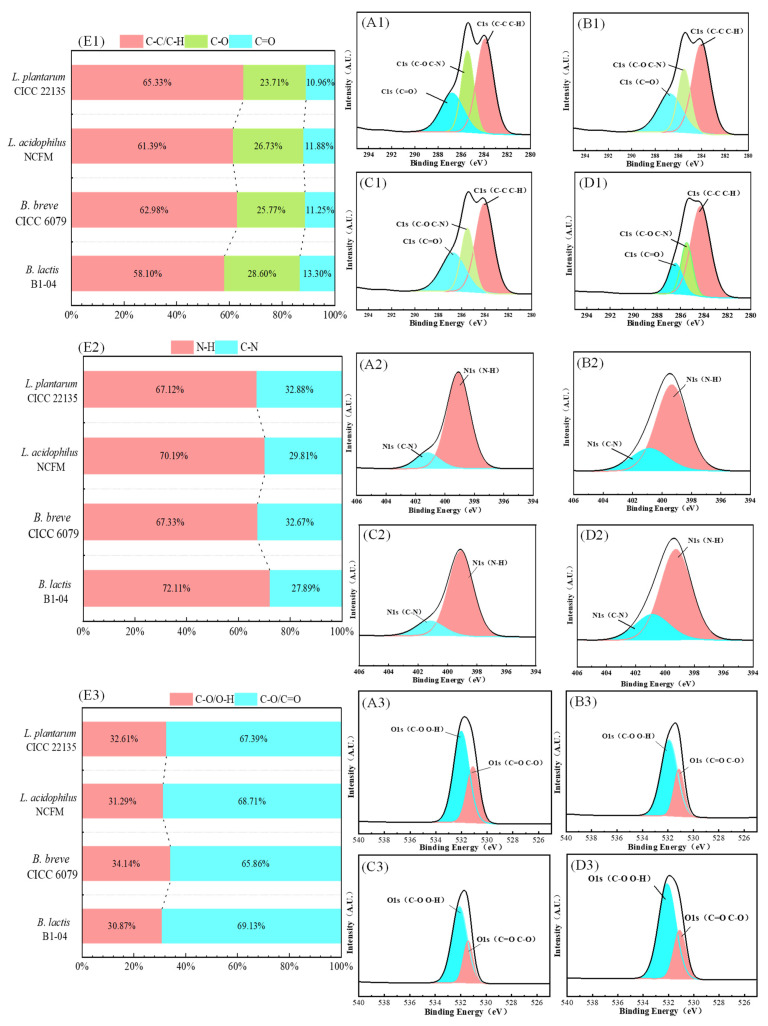
(**A1**–**D1**) C spectrum of four selected LAB-based PGNs. ((**A1**): *B. lactis* B1-04; (**B1**): *B. breve* CICC 6079; (**C1**): *L. acidophilus* NCFM; (**D1**): *L. plantarum* CICC 22135). (**E1**) The relative content of carbon-containing groups in the tested LAB stain PGN. (**A2**–**D2**) N spectrum of four selected LAB-based PGNs. ((**A2**): *B. lactis* B1-04; (**B2**): *B. breve* CICC 6079; (**C2**): *L. acidophilus* NCFM; (**D2**): *L. plantarum* CICC 22135). (**E2**) The relative content of nitrogen-containing groups in the tested LAB stain PGN. (**A3**–**D3**) O spectrum of four selected LAB-based PGNs. ((**A3**): *B. lactis* B1-04; (**B3**): *B. breve* CICC 6079; (**C3**): *L. acidophilus* NCFM; (**D3**): *L. plantarum* CICC 22135). (**E3**) The relative content of oxygen-containing groups in the tested LAB stain PGN.

The peak C1s at 284.8 eV is used as a reference for all binding energies of the spectrograms for surface charge calibration, as seen in Figure 7A1–D1. The C1s peaks of LAB-based PGN from the four tested strains could be identified as three carbon bonds, namely the C-C or C-H single bond, represented by binding energy from 284.28 to 284.45 eV [42], the C-O single bond, illustrated by binding energy from 286.75 to 286.92 eV [43], and the C=O double bond, located from 287.56 to 287.86 eV [44]. Thus, the relative content of each specific group from the LAB-based PGNs can be directly determined by the peak area of its XPS spectrum [36]. In this study, the relative contents of C-C/C-H groups in aliphatic or amino acid side chains were 65.33% for *L. plantarum* CICC 22135, 62.98% for *B. breve* CICC 6079, 61.39% for *L. acidophilus* NCFM, and 58.10% for *B. lactis* B1-04 according to their spectral peak information. The relative contents of C-O groups in protein amides and C=O in a ketone or carboxyl groups were 28.60% and 13.30% for *B. lactis* B1-04, 26.73% and 11.88% for *L. acidophilus* NCFM, 25.77% and 11.25% for *B. breve* CICC 6079, and 23.71% and 10.96% for *L. plantarum* CICC 22135 (see Table 3). Form Figure 7E1, we can learn the relative contents of the C-O single bond and C=O bond of PGN in four strains, among which *B. lactis* B1-04 had the highest, followed by *L. acidophilus* NCFM, *B. breve* CICC 6079, and *L. plantarum* CICC 22135. Moreover, it is interesting to find the higher the relative content of C-O single bonds of saccharides (C-O, C-O-H, C-O-C) and C=O bonds in LAB-based PGNs, the higher the adsorption rate of PGN to AA/HMF. Clearly, the C-O single bond and C=O double bond in LAB-based PGNs play an important role in the AA/HMF binding.

#### 3.4.3. N Spectrum

The data from Figure 7A2–D2 show that the N1s peaks of LAB-based PGNs from the four tested strains could be identified as two nitrogen bonds, namely the amide bond C-N in protein, illustrated by binding energy from 400.01 to 401.05 eV [45], and the N-H group, represented at the binding energy from 399.27 to 399.34 eV [46]. Moreover, the relative content of amide C-N in the protein was 32.88% for *L. plantarum* CICC 22135, 32.67% for *B. breve* CICC 6079, 29.81% for *L. acidophilus* NCFM, and 27.89% for *B. lactis* B1-04. The relative content of the amino N-H group was 72.11% for *B. lactis* B1-04, 70.19% for *L. acidophilus* NCFM, 67.33% for *B. breve* CICC 6079, and 67.12% for *L. plantarum* CICC 22135 (see Table 4). From Figure 7E2, we can learn the relative contents of the N-H bond of PGN in the four strains, among which *B. lactis* B1-04 had the highest content, followed by *L. acidophilus* NCFM, *B. breve* CICC 6079, and *L. plantarum* CICC 22135. Moreover, it is interesting to find the higher the relative content of the N-H bond in LAB-based PGNs, the higher the adsorption rate of PGN to AA/HMF. Obviously, the N-H bond in LAB-based PGNs plays an important role in the AA/HMF-binding.

#### 3.4.4. O Spectrum 

As shown in Figure 7A3–D3, the O1s peaks of LAB-based PGN from the four tested strains could be classified into two oxygen bonds, namely the C-O and O-H bond (oxygen of alcohols and acetals), represented by binding energy from 532.15 to 532.37 eV [47], and the C-O and C=O bond (oxygen in carboxyl, carbonyl, ester, or amide), illustrated by the binding energy from 531.18 to 531.56 eV [46]. Moreover, the relative content of the C-O and O-H group in the carbohydrate hydroxyl was 34.14% for *B. breve* CICC 6079, 32.61% for *L. plantarum* CICC 22135, 31.29% for *L. acidophilus* NCFM, and 30.87% for *B. lactis* B1-04. The relative content of the C-O and C=O bond of saccharides was 69.13% for *B. lactis* B1-04, 68.71% for *L. acidophilus* NCFM, 67.39% for *L. plantarum* CICC 22135, and 65.86% for *B. breve* CICC 6079 (see Table 5). Unlike the XPS C1s and N1s, however, analysis of XPS O1s did not find a significant correlation between the binding capacity of LAB-based PGNs to AA/HMF and the specific groups of O-H, C-O, and C=O. A possible reason for this to occur might be that the binding energies of the C-O group presented by the O spectrum are between 532.15 and 532.37 eV for both O-H and C-O groups, and between 531.18 and 531.56 eV for both C=O and C-O groups. Thus, it is not easy to definitely distinguish the O-H group from the C=O group in terms of the two binding energies. As a result, significant correlations were not found between the binding capacity of LAB-based PGNs to AA/HMF and the specific groups of O-H, C-O, and C=O.

## 4. Discussion

Generally, interaction energy is usually used to describe the adsorption performance between molecules [48]. Previous studies have indicated that interaction energy is mainly responsible for the adsorption behavior of LAB-based PGNs of the selected strains to mutagenic compounds [19]. To investigate the ability of the PGN of each tested strain to bind AA or HMF, an in silico study showing the interaction energy between the PGN of each selected strain and AA or HMF was carried out, as listed in Table 1. Our in silico results showed that the interaction energy between the PGN and AA or HMF was strain-specific. Among the four selected bacterial strains, the highest interaction energy was obtained between *Bifidobacterium lactis* B1-04 PGN and AA or HMF, followed by *Lactobacillus acidophilus* NCFM, *Bifidobacterium breve* CICC 6079, and *Lactobacillus plantarum* CICC 22135. As confirmed by our in vitro results, indeed, strain *Bifidobacterium lactis* B1-04 PGN bound the most AA (28.7%) and HMF (48.0%) of the strains (Figure 1A,B). A previous study showed that the ability of LAB-based PGN in removing AA significantly differed, depending on the tested strains, i.e., *Lactobacillus plantarum* 1.0065, *Lactobacillus casei* ATCC393, *Lactobacillus acidophilus* KLDS1.0307, and *Lactobacillus thermophilus* KLDS1.0316 [24]. The work of Albedwawi et al. found that among the 40 tested strains, the best reduction rates of the amide (AA) were 35% for *B. breve* and 36% for *L. acidophilus*, depending on the species and strain used [25]. This meant that the adsorption efficiency of each tested strain towards AA or HMF might be greatly attributed to its PGN. A similar study conducted by Zhao et al. indicated that the interaction energy, including van der Waals force, electrostatic force, and hydrogen bond force, is responsible for the adsorption of the selected LAB’s PGN towards DBP [49], which is consistent with our current results. Moreover, Song et al. stated that the greater the interaction energy, making the molecules closer together, the smaller the fractional free volume [50]. Thus, we calculated the interaction energy as well as the fractional free volume between the PGN and AA/HMF. It was found that the larger the interaction energy between the PGN and AA/HMF, the smaller the FFV (Figure 5C,D), indicating that the interaction energy between them becomes stronger with a higher binding of PGN to AA/HMF. Similarly, the study of Zhao et al. (2021) showed that the lower the FFV, the higher the interaction energy of LAB-based PGN binding DBP, thus producing a higher adsorption rate [19]. Thus, the strength of the interaction energy between the PGNs and AA/HMF is likely a good indicator, showing the ability of each LAB strain to bind the mutagenic chemicals.

Many studies have suggested that the groups C-O, C=O, N-H, and O-H from LAB’s PGN are key sites involved in the adsorption of different chemicals like DBP [19], BPA [51], and DEHP [20]. However, how these LAB-based PGN groups affect the ability of each tested strain to bind AA/HMF is still unknown. To clarify the adsorption action of the mode of these key sites, the roles of these functional groups from *B. lactis* strain B1-04 PGN binding with AA/HMF were investigated via FTIR, XRD, AFM, and SEM. It was seen that significant changes during the adsorption process of strain B1-04 PGN towards AA/HMF occurred in the groups C-O, C=O, N-H, and O-H, as observed in the FTIR data (Figure 3C). Moreover, data from XRD, as shown in Figure 3B, showed new acute crystal peaks, which appeared after strain B1-04 PGN bound AA/HMF, indicating the original crystal structure was changed. Morphologically, it was observed that the cell wall surface of strain B1-04 was covered with an adsorption layer with a thickness of 50 to 200 nm after the bacterium bound AA/HMF (Figure 2). The cell wall surface of strain B1-04 was seen to become rougher than the control due to the presence of adsorbed AA/HMF (Figure 3A). Molecular docking between *B. lactis* strain B1-04 PGN and AA/HMF shows that nine binding sites are formed after the bacterium binds the two chemicals (Figure 3D). The binding site of strain B1-04 PGN acts on AA in the order of O3, N2, N1, H2, N3, O2, O4, O1, H1 (Figure 3E), and on HMF in the order of H2, O3, O2, H1, O1, N1, O4, N3, N2 (Figure 3F). Meanwhile, the groups C-O, C=O, N-H, and O-H from strain B1-04 PGN change markedly after binding. A recent study showed that the groups C-O, N-H, O-H, and C-N from *L. acidophilus* NCFM PGN are the main components responsible for binding DBP [19]. Goyal et al. revealed the importance of the functional groups C=O, N-H, C-N, and C-O from *L. rhamnosus* GG and *L. acidophilus* MTCC 25433 PGNs played major roles in removing DEHP [20]. Similarly, C-O, C-O, and N-H have been suggested as the main functional groups of the PGN from *B. breve* and *L. acidophilus* to adsorb AA [25]. Clearly, the specific groups of LAB-based PGN, including C-O, C=O, N-H, and O-H, are likely the main components responsible for the adsorption of AA/HMF. 

Generally, the LAB cell wall PGN is a large molecule with a dense network structure, which is polymerized by a disaccharide unit, tetrapeptide tail, and peptide bridge. The structure of PGN in bacterial cell walls is influenced by the different compositions of the tetrapeptide tail and the peptide bridge [52]. However, it is not clear how the structural differences in the LAB-based PGN affect the ability of the tested LAB strains to remove harmful chemicals regarding the content and types of the functional groups distributed on PGN that bind with AA/HMF. XPS, as a powerful technique, has been used to analyze the chemical properties of the bacterial cell surface, including Gram-negative [53] and Gram-positive [36] properties. Ma et al. obtained the relative content of specific functional groups from different samples by analyzing the elemental peaks of XPS C1s, N1s, and O1s peaks [37]. In our case, XPS was explored to investigate the relationship between the relative content of the major specific groups from the four tested LAB PGNs and four selected LAB PGNs binding to AA/HMF. It was observed that significant peaks appeared in the spectra of carbon, nitrogen, and oxygen after LAB strains’ PGNs bound AA/HMF (see Figure 6). Furthermore, we discovered the content of specific groups on LAB-based PGNs affects its binding ability towards AA/HMF to some extent. The higher the relative contents of C-O, C=O, and N-H of specific groups distributed on LAB-based PGNs, the higher the adsorption rate of AA/HMF. It is interesting to note the relative content of C-C and C-N groups of the specific groups on LAB-based PGNs were opposite to the adsorption rates of AA/HMF. To our knowledge, this is the first report confirming the binding effect of LAB-based PGN on AA/HMF and that the functional groups C-O, C=O, and N-H in the cell wall PGN play the main role in the adsorption process of PGN toward AA/HMF. In light of these findings, the PGN obtained from the selected LAB strains with a strong adsorption effect on AA/HMF is expected to be used for the removal of AA/HMF in hot processed foods.

## 5. Conclusions

As mentioned in previous studies, LAB-original PGNs play a significant role in adsorbing AA/HMF via their interaction energy and specific groups. Importantly, the adsorption efficiency of the tested lactic acid bacteria to AA/HMF is strain-dependent and is especially related to the specific groups of their PGNs, including C-O, C=O, and N-H groups. Among the four selected bacterial strains, *B. lactis* B1-04 bound the most AA or HMF, followed by *L. acidophilus* NCFM, *B. breve* CICC 6079, and *L. plantarum* CICC 22135, depending on the difference in the relative content of their specific groups distributed on PGNs. In summary, the LAB-based PGN is a very effective biological means to adsorb AA/HMF from the medium and provides a solid theoretical basis for the subsequent use of LAB-based PGN to remove (or alleviate) AA/HMF. Further studies are needed to elucidate the ability of LAB-based PGN to adsorb AA/HMF in the context of in vivo digestion, thus providing useful considerations for the food and healthcare industries.

## Figures and Tables

**Table 1:** Interaction energy of LAB-based PGNs binding with AA/HMF.

Strains	Simulation System	Total Energy (kcal/mol)
	E_AA_	24.613
	E_HMF_	28.193
*B. lactis*	E_PGN1_	571.464
	E_PGN1+AA_	843.164
	E_PGN1+HMF_	892.950
	△E_A1_	247.087
	△E_H1_	293.293
*B. breve*	E_PGN2_	482.068
	E_PGN2+AA_	707.387
	E_PGN2+HMF_	768.930
	△E_A2_	200.706
	△E_H2_	258.669
*L. acidophilus*	E_PGN3_	496.291
	E_PGN3+AA_	743.481
	E_PGN3+HMF_	828.534
	△E_A3_	222.577
	△E_H3_	273.210
*L. plantarum*	E_PGN4_	530.694
	E_PGN4+AA_	737.666
	E_PGN4+HMF_	768.596
	△E_A4_	182.359
	△E_H4_	247.675

**Table 2:** The interaction energy and fractional free volume of AA/HMF bound by four selected LAB-based PGNs.

Bacterial Species	Interaction Energy I ^(1)^ (kcal/mol)	FFVⅠ(%)	Interaction Energy II ^(2)^ (kcal/mol)	FFV II (%)
*B. lactis* B1-04	247.09	24.12	293.29	22.10
*B. breve* CICC 6079	200.71	29.81	258.67	27.88
*L. acidophilus* NCFM	222.57	27.09	273.21	26.11
*L. plantarum* CICC 22135	182.36	33.67	247.68	28.12

^(1)^ I represents the simulation system of LAB-based PGN bound to AA. ^(2)^ II represents the simulation system of LAB-based PGN bound to HMF.

**Table 3:** C spectrum information of LAB-based PGNs.

Peak	Binding Energy (eV)	Proportion of Fitted Peak Area (%)				Group
		*B. lactis* B1-04	*B. breve* CICC 6079	*L. acidophilus* NCFM	*L. plantarum* CICC 22135	
C1	284.28–284.45	1,935,134 (58.1%)	20,984,085 (62.98%)	20,454,318 (61.39%)	217,670,731 (65.33%)	C-C/C-H
C2	286.75–286.92	9,529,133 (28.6%)	8,586,216 (25.77%)	8,906,075 (26.73%)	7,899,851 (23.71%)	C-O
C3	287.56–287.86	4,431,380 (13.3%)	3,748,348 (11.25%)	3,958,255 (11.88%)	3,651,724 (10.96%)	C=O

**Table 4:** N spectrum information of LAB-based PGNs.

Peak	Binding Energy (eV)	Proportion of Fitted Peak Area (%)				Group
		*B. lactis* B1-04	*B. breve* CICC 6079	*L. acidophilus* NCFM	*L. plantarum* CICC 22135	
N1	399.27–399.34	41,658 (72.11%)	38,897 (67.33%)	40,549 (70.19%)	38,776 (67.12%)	N-H
N2	400.01–401.05	116,112 (27.89%)	18,874 (32.67%)	17,221 (29.81%)	18,995 (32.88%)	C-N

**Table 5:** O spectrum information of LAB-based PGNs.

Peak	Binding Energy (eV)	Proportion of Fitted Peak Area (%)				Group
		*B. lactis* B1-04	*B. Breve* CICC 6079	*L. acidophilus* NCFM	*L. plantarum* CICC 22135	
O_1_	532.15–532.37	77,819 (30.87%)	86,062 (34.14%)	78,878 (31.29%)	82,205 (32.61%)	-O/O-H
O_2_	531.18–531.56	174,267 (69.13%)	166,024 (65.86%)	173,209 (68.71%)	169,881 (67.39%)	C-O/C=O

## Data Availability

The data are confidential.
